# Draft Genome Sequences of Two Isolates of the *Roseobacter* Group, *Sulfitobacter* sp. Strains 3SOLIMAR09 and 1FIGIMAR09, from Harbors of Mallorca Island (Mediterranean Sea)

**DOI:** 10.1128/genomeA.00350-14

**Published:** 2014-05-22

**Authors:** Maria Mas-Lladó, Joana Maria Piña-Villalonga, Isabel Brunet-Galmés, Balbina Nogales, Rafael Bosch

**Affiliations:** Microbiologia, Departament de Biologia, Universitat de les Illes Balears (UIB), Palma de Mallorca, Spain

## Abstract

We present the draft genome sequences of two isolates of the *Roseobacter* lineage, 3SOLIMAR09 and 1FIGIMAR09, which were obtained from harbors of Mallorca Island, Spain, and are affiliated with the *Sulfitobacter* genus. Both isolates harbor the complete gene set for protocatechuate catabolism and incomplete pathways for several additional monoaromatic compounds.

## GENOME ANNOUNCEMENT

The *Roseobacter* lineage is a diverse group within *Alphaproteobacteria* that can represent up to 20% of marine microbial communities (1). The catabolism of organic compounds (including aromatic hydrocarbons), sulfur and carbon monoxide oxidation, and aerobic anoxygenic photosynthesis are some important traits that can be found in this group (2–4). Due to their role in biogeochemical marine cycles, the interest in isolating and describing new members of this lineage has increased in recent years. In fact, >40 genomes have been sequenced, including two members of the *Sulfitobacter* genus (EE-36 and NAS-14.1).

Strains 3SOLIMAR09 and 1FIGIMAR09 were isolated in March 2009 by plating surface water samples from the Sóller and Cala Figuera harbors (Mallorca Island, Spain), respectively, on marine agar (5). According to 16S rRNA gene sequence analysis, they were affiliated with the genus *Sulfitobacter*: 3SOLIMAR09 was found to be related to *Sulfitobacter pontiacus* (99.86% identity with strain DSM 10014^T^) and 1FIGIMAR09 to *Sulfitobacter mediterraneus* (99.79% identity with strain DSM 12244^T^). Neither the *S. pontiacus* nor *S. mediterraneus* type strain genomes have been sequenced.

Genome sequencing was done using Illumina technology. The reads were assembled using Newbler version 2.9. Genome annotation, comparison, and analysis were done using the Prokaryotic Genome Automatic Annotation Pipeline (PGAAP) at NCBI (http://www.ncbi.nlm.nih.gov/genome/annotation_prok/), the automatic annotation server at KEGG (6), ISfinder (7), and the JSpecies (8) program.

The average nucleotide identity based on BLAST (ANIb) (8) suggested that strains 3SOLIMAR09 and EE-36 (9) belong to the same species (97.6% genome identity). The ANIb index between the isolates 3SOLIMAR09 and 1FIGIMAR09 (71.1% of genome identity) indicated that they belong to different *Sulfitobacter* species.

The draft genome of 3SOLIMAR09 (25 contigs of >500 bp) is 3,453,172 bp in length (56-fold coverage) and has 60.35 mol% G+C, 3,284 coding sequences (CDSs), and 40 tRNAs. The rRNA operon was split in two contigs; their depths of coverage (223- and 209-fold) suggested the presence of four rRNA operons. Seventeen plausible transposases belonging to 6 different insertion sequence (IS) families, as well as 4 integrase-like proteins, were identified.

The draft genome of 1FIGIMAR09 was assembled into 55 contigs (88-fold coverage). It is 3,861,701 bp in length and has 58.43 mol% G+C content. It harbors 3,649 CDSs and 39 tRNAs. The entire rRNA operon was coded in a single contig. Its 206-fold coverage suggested the presence of two rRNA operons. Ten integrase-like proteins and 9 plausible transposases from 5 different IS families were also predicted.

The putative genes for major metabolic pathways (i.e., tricarboxylic acid [TCA] cycle and pentose phosphate pathway) were predicted in both genomes. Both genomes lacked genes for a photosynthetic reaction center, indicating that the isolates were unable to perform aerobic anoxygenic photosynthesis. In relation to their chemolithotrophic capabilities, both genomes harbor all putative genes for sulfite oxidation (*sox* system), but only strain 1FIGIMAR09 has the entire *coxSLM* operon that encodes carbon monoxide dehydrogenase (strain 3SOLIMAR09 lacks the *coxL* gene). Finally, both strains harbor a complete aromatic hydrocarbon degradation pathway, the protocatechuate branch of the β-ketoadipate pathway. Both strains also harbor genes related to the catabolism of homoprotocatechuate, homogentisate, gentisate, and phenylacetate, but those metabolic pathways seemed to be incomplete.

### Nucleotide sequence accession numbers.

The whole-genome shotgun projects of strains 3SOLIMAR09 and 1FIGIMAR09 have been deposited at DDBJ/EMBL/GenBank under accession no. AXZR00000000 and JEMU00000000, respectively. The versions described in this paper are AXZR01000000 and JEMU01000000.

## References

[1] Buchan A González JM Moran MA 2005 Overview of the marine *Roseobacter* lineage. Appl. Environ. Microbiol. 71:5665–5677. 10.1128/AEM.71.10.5665-5677.2005 16204474 PMC1265941

[2] Buchan A González JM 2010 Roseobacter, p 1335–1343 *In* Timmis KN (ed), Handbook of hydrocarbon and lipid microbiology. Springer-Verlag, Berlin, Germany

[3] Wagner-Döbler I Biebl H 2006 Environmental biology of the marine *Roseobacter* lineage. Annu. Rev. Microbiol. 60:255–280. 10.1146/annurev.micro.60.080805.142115 16719716

[4] Newton RJ Griffin LE Bowles KM Meile C Gifford S Givens CE Howard EC King E Oakley CA Reisch CR Rinta-Kanto JM Sharma S Sun S Varaljay V Vila-Costa M Westrich JR Moran MA 2010 Genome characteristics of a generalist marine bacterial lineage. ISME J 4:784–798. 10.1038/ismej.2009.150 20072162

[5] Piña-Villalonga JM 2012 Diversidad e importancia ecológica del grupo *Roseobacter* en aguas costeras sometidas a impacto antropogénico. Ph. D. thesis University of Balearic Islands (UIB), Palma de Mallorca, Spain (In Spanish)

[6] Moriya Y Itoh M Okuda S Yoshizawa AC Kanehisa M 2007 KAAS: an automatic genome annotation and pathway reconstruction server. Nucleic Acids Res. 35:W182–W185. 10.1093/nar/gkm321 17526522 PMC1933193

[7] Siguier P Perochon J Lestrade L Mahillon J Chandler M 2006 ISfinder: the reference centre for bacterial insertion sequences. Nucleic Acids Res. 34:D32–D36. 10.1093/nar/gkj014 16381877 PMC1347377

[8] Richter M Rosselló-Móra R 2009 Shifting the genomic gold standard for the prokaryotic species definition. Proc. Natl. Acad. Sci. U. S. A. 106:19126–19131. 10.1073/pnas.0906412106 19855009 PMC2776425

[9] González JM Whitman WB Hodson RE Moran MA 1996 Identifying numerically abundant culturable bacteria from complex communities: an example from a lignin enrichment culture. Appl. Environ. Microbiol. 62:4433–4440 8953714 10.1128/aem.62.12.4433-4440.1996 PMC168269

